# The role of ultraviolet reflectance and pattern in the pollination system of *Hypoxis camerooniana* (Hypoxidaceae)

**DOI:** 10.1093/aobpla/plz057

**Published:** 2019-09-23

**Authors:** Yannick Klomberg, Raissa Dywou Kouede, Michael Bartoš, Jan E J Mertens, Robert Tropek, Eric B Fokam, Štěpán Janeček

**Affiliations:** 1 Department of Ecology, Faculty of Science, Charles University, Viničná 7, 12844 Prague, Czechia; 2 Department of Zoology and Animal Physiology, Faculty of Science, University of Buea, PO Box 63 Buea, Cameroon; 3 Institute of Botany, Czech Academy of Sciences, Dukelská 135, 37901 Třeboň, Czechia; 4 Institute of Entomology, Biology Centre, Czech Academy of Sciences, Branišovská 31, 37005 České Budějovice, Czechia

**Keywords:** Afromontane grasslands, floral traits, foraging behaviour, Mount Cameroon National Park, pollination interactions, UV manipulation

## Abstract

Apart from floral morphology and colours perceived by the human eye, ultraviolet (UV) reflectance acts as an important visual advertisement of numerous flowering plant species for pollinators. However, the effect of UV signalling on attracting pollinators of particular plant species is still insufficiently studied, especially in the Afrotropics. Therefore, we studied the pollination system of *Hypoxis camerooniana* in montane grasslands of Mount Cameroon, West/Central Africa. We focused mainly on the effects of the flowers’ UV reflectance on its visitors. We experimentally removed UV reflection from petals either completely or partially. Thereafter, flower visitors were recorded and pistils were collected post-flowering to quantify germinated pollen tubes per treatments. The most important visitors were bees, followed by flies. Due to their contacts with reproductive organs bees are considered as the primary pollinators. Visitation rates were lower when UV reflectance was completely removed, whereas the decrease of frequency on half-treated flowers did not differ significantly from control treatments. The complete removal of UV also affected bees’ landing behaviour, but not that of flies. We showed that the presence of UV reflectance is more important than UV pattern for bees visiting flowers of *H. camerooniana*. We hypothesize that exploiting all flowers irrespective of their pattern can be more efficient for pollinators in the open grasslands of high altitudes to spot these relatively scarce flowers by their UV reflectance. Furthermore, we highlight the necessity of both experimental and natural controls in similar studies to control for additional effects of the used UV manipulations.

Many plants advertise their flowers with UV reflectance visible to their insect visitors. By manipulating the UV reflectance and pattern of *Hypoxis camerooniana* in the Afromontane grasslands of Mount Cameroon, we have shown how crucial it is for the predominant visitor, bees. Both bees' preferences for flowers and their behaviour during visits are influenced by changes in UV reflectance. However, the presence of some UV signal is more important than the specific pattern. Especially in montane grasslands with higher UV irradiation, the UV floral colours are important for recognition of flowers by potential pollinators.

## Introduction

Unlike humans, many insect pollinators are sensitive to the ultraviolet (UV) part of the electromagnetic light spectrum in addition to the visible spectrum (Briscoe and Chittka 2001). Ultraviolet light is reflected by flowers of ~25 % of angiosperms, with the highest reflectance found in plant species with yellow flowers (Chittka *et al.* 1994; Papiorek *et al.* 2016). Consequently, the UV vision helps floral visitors in recognition of individual flowers of such plants which differ in their UV colouration from other plants in the community (Johnson and Andersson 2002). To increase distinction by certain groups of pollinators, some flowers create a contrasting pattern of UV absorbance and reflectance on the surface of their petals, whereas others contrast petals and reproductive parts by an inverse pattern of absorbance and reflectance of UV light. Floral guides (Penny 1983; Dinkel and Lunau 2001; Lunau 2006; Papiorek *et al.* 2016) and the so-called bullseye patterns (Lunau 1992a; Koski and Ashman 2014, 2015a, b), which has reflecting apices and absorbing bases of petals, are among the most commonly known examples of this phenomenon. These UV patterns are believed to improve the identification of the landing and/or foraging parts of flowers, or mimic such parts to the pollinator (Lunau *et al.* 2017). Their importance was shown in numerous studies revealing the influence of UV patterns on pollinator visitation preferences (e.g. Burr *et al.* 1995; Campbell *et al.* 2010; Horth *et al.* 2014; Koski and Ashman 2014; Peterson *et al.* 2015) and behaviour (Hansen *et al.* 2012).

The specific colour vision, which includes UV, of some insects and spectral properties of flowers have evolved into mutualistic relationships between plants and their pollinators. One of the best understood systems of vision is that of bees (Dyer *et al.* 2015). von Helversen (1972) measured the capability of honeybees to distinguish colours and showed that bees best discriminate wavelengths at ~400 and 500 nm where the spectral sensitivity curves of UV, blue and green photoreceptors overlap. Peitsch *et al.* (1992) quantified this further, showing the trichromatic vision of 43 different taxa of hymenopterans with the sensitivity peak of UV light at 340 nm, blue light at 430 nm and yellow light at 535 nm. Some specific colour patterns of flowers, such as floral guides, are of such importance for bee flower recognition that they were included into *melittophily*, i.e. the pollination syndrome related to bees (Faegri and van der Pijl 1979; Lunau 1992a; Fenster *et al.* 2004; Willmer 2011). However, the utility of these syndromes has been questioned recently (Ollerton *et al.* 2009), and some authors started using more precise systems, such as looking at single explanatory traits (e.g. Stang *et al.* 2009) or buzz pollination (e.g. De Luca and Vallejo-Marín 2013). The importance of UV patterns for bees was confirmed by a decreased frequency of flower visitation by different bee species after elimination of the UV reflection from petals of various plant species (Johnson and Andersson 2002; Peter and Johnson 2008; Welsford and Johnson 2012; Rae and Vamosi 2013; Brock *et al.* 2016).

Nevertheless, floral colour evolution has been influenced by numerous other factors (Grimaldi 1999; Friis *et al.* 2011; Song *et al.* 2015), such as floral defence against solar radiation (Robberecht and Caldwell 1978; Koski and Ashman 2015a). It was shown that the absorbance of UV-A by plant tissues can be related to plant protection against harmful UV-B radiation (Robberecht and Caldwell 1978; Caldwell *et al.* 1983). The importance of UV colour reflectance and absorbance can be manifested along the gradient of UV irradiation, e.g. towards high altitudes and the equator (Johnson *et al.* 1976; Beckmann *et al.* 2014; Koski and Ashman 2015a). It was shown that UV irradiance as the selection agent affects the size of the UV-absorbing floral centre (bullseye), with increases towards the equatorial ecosystems and along altitudinal gradients as well (Koski and Ashman 2015a, b). Despite long-term research on floral UV signalling many questions remain unanswered. For example, it is unclear if the ability of pollinators to recognize the flower is caused by any UV-reflecting area on the flower or if it is related to specific UV patterns. Additionally, we only have limited knowledge on how the common experimental manipulation using UV-absorbing creams (e.g. Johnson and Andersson 2002; Peter and Johnson 2008; Welsford and Johnson 2012; Rae and Vamosi 2013) generally affects the natural (i.e. unmanipulated) pollination system of the studied plant species.

**Figure 1.**
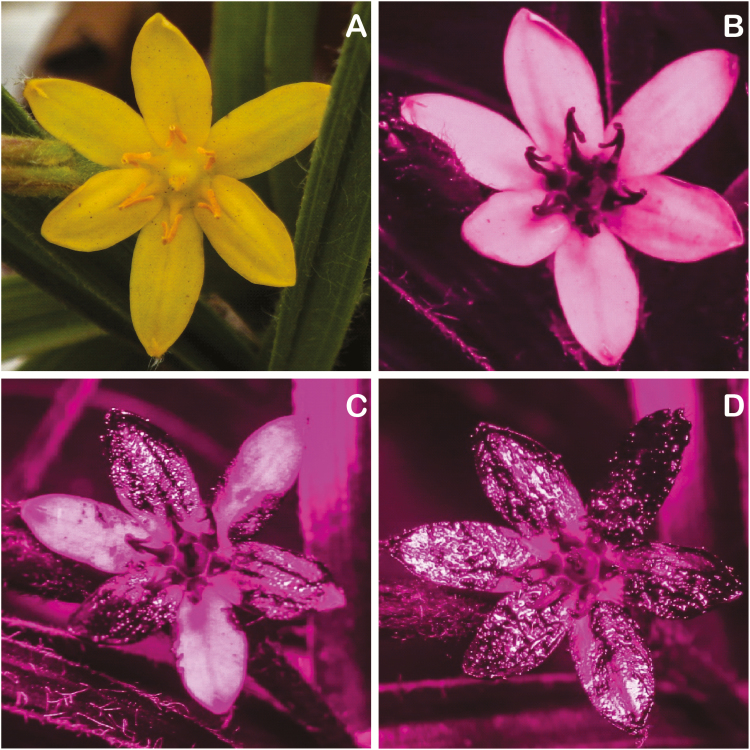
A flower of *H. camerooniana*: (A) a normal photograph, (B) a UV photograph of a non-manipulated flower, (C) a UV photograph with half absorbent cream treatment (*UV*_*50 %*_), (D) a UV photograph with full absorbent cream (*UV*_*100 %*_) treatment.

To study the role of UV reflection in pollination we selected *Hypoxis camerooniana* (Hypoxidaceae) as model species. *Hypoxis camerooniana* is endemic to the mountains of south-western Nigeria and western Cameroon (Hutchinson *et al.* 1968) and therefore, better understanding its pollination system can help to better focus potential conservation efforts for both plant and its pollinators. Due to growing at low latitudes and high altitudes (above 2000 m a.s.l. on Mount Cameroon), *H. camerooniana* is exposed to intensive UV irradiance (Johnson *et al.* 1976; Beckmann *et al.* 2014). It has yellow UV-reflecting petals (Fig. 1) and yellow UV-absorbing anthers, consequently creating a contrasting central pattern in the flower. Firstly, we aimed to gain insights in the unknown pollination system of this endemic plant. Secondly, to study the role of UV colour on the visitation frequency, behaviour and pollination success of *H. camerooniana*, we used the same approach as Johnson and Andersson (2002) by manipulating flowers with an UV-absorbing cream either by complete removal of the UV reflectance, or by maintaining the UV reflectance on half of petals, i.e. changing the UV pattern (Fig. 1). Our study extends the previous work of, e.g., Johnson and Andersson (2002) by including a natural unmanipulated control to test the influence of the experimental treatments on the flower visitation frequency.

## Materials and Methods

### Study locality

This study was carried out at the montane grasslands above Mann’s Spring (~2250 m a.s.l.) on Mount Cameroon, the highest mountain in western and central sub-Saharan Africa (4095 m a.s.l.; Cable and Cheek 1998). It is situated in the Southwest region of Cameroon (4.203°N and 9.170°E), offering a wide range of habitats (see Cable and Cheek 1998) and hosting a great biodiversity including endemics with exceptional ecological features (Bergl *et al.* 2007). Especially ecotones along the timberline, found also at Mann’s Spring, harbour many species which are not found elsewhere on the mountain

### Study plant

The genus *Hypoxis* contains an estimated 90 species in Africa, North and South America, South-Eastern Asia and Australia, with the centre of diversity in Southern Africa (Singh 1999). These plants are characterized by their bright yellow flowers, lanceolate and densely hairy leaves. They are weak competitors and thus grow mostly in places with low vegetation cover. *Hypoxis camerooniana* (Hypoxidaceae) is a perennial pyrophytic herb, restricted to high elevations of the Cameroonian Volcanic Line (Cable and Cheek 1998; GBIF Secretariat 2019). Its leaves are tristichous, 50 cm long and 0.5–2 cm wide, covered with golden hairs, recurved and ± prostrate to erect. On a scape up to 25 cm tall 5–7 flowers can be found (African Plant Database 2019). On Mount Cameroon, we always observed only 1 or 2 active flowers per plant. The flowers have a short lifespan, they open at daybreak and usually last 1 day or less.

*Hypoxis* species are used across Africa as traditional medicine and were reported to have a wide spectrum of pharmacological properties (Ncube *et al.* 2013). *Hypoxis hemerocallidea* has already been known for its strong UV reflectance and due to its relatively robust flowers was used for manipulative experiments with UV-absorbing cream (Johnson and Andersson 2002). Outside of the two sweet-smelling species *H. fischerii* var. *zernyi* and *H. goetzei* East African *Hypoxis* flowers appeared to be without scent (Wiland-Szymańska 2009). So far, no nectar has been found in any *Hypoxis* species (Johnson and Andersson 2002; Rudall 2002; Ren *et al.* 2019).

Some studied *Hypoxis* species are pollinated by solitary bees and honeybees (Singh 1999; Johnson and Andersson 2002), while the autogamous *H. decumbens* attracts ‘generalist insects, like dipterans’ (Raimúndez and Ramirez 1998). Furthermore, pollen- and tepal-feeding beetles were observed in Southern and Eastern Africa (Steiner 1998; Wiland-Szymańska 2009). No data on pollination and visitation of *H. camerooniana* exist.

### UV manipulation

The study was carried out in October and November 2016. To manipulate the UV patterns on flowers, we followed the approach of Johnson and Andersson (2002). The studied specimens of *H. camerooniana* were randomly selected in the study area. At daybreak, just after opening of selected buds, four different treatments were applied: (i) *UV*_*100 %*_*treatment*, i.e. complete removal of UV reflectance from the flower using UV cream on all petals of the flower; (ii) *FAT*_*100 %*_*treatment*, i.e. a control for *UV*_*100 %*_, all petals were covered with duck preen gland fat, a non-UV-absorbing cream compound; (iii) *UV*_*50 %*_*treatment*, i.e. the UV cream was used on three out of six petals, covering every second petal; and (iv) *FAT*_*50 %*_*treatment*, i.e. a control for *UV*_*50 %*_, every second petal was treated with the non-UV-absorbing compound. Both UV cream and non-UV-absorbing cream were applied carefully using cotton swabs. Besides these four treatments, natural (*Natural control*) non-manipulated flowers were studied to control for the effect of any treatment on flowers. The UV-absorbing chemicals were equal amounts of Parsol 1789® (butyl methoxydibenzoylmethane) and Parsol MCX® (ethylhexyl methoxycinnamate) dissolved in the duck preen gland fat as a solvent (at 40:60 w/w) by gentle heating (Andersson and Amundsen 1997; Johnson and Andersson 2002). On each day of the experiment, 10 plant specimens were selected in the grasslands and randomly treated, two replicates of each treatment per day, resulting in a total of 50 experimental plants. When two flowers were found on a single experimental plant, we applied the same treatment for both. Each experimental flower was recorded by a security camera (VIVOTEK IB8367-T with IR night vision) for 24 h following Mertens *et al.* (2018); however, most of the flowers were short-lived and closed at the beginning of the night after ~12 h of recording. Due to the short lifespan of flowers, all their visitors were certainly observed. Afterwards the recordings were watched, and all floral visitors were noted. Besides arrival of visitors we also identified them to the most detailed taxon level as possible, and we noted their behaviour (both landing behaviour and activity after landing, e.g. feeding on pollen) and touches to reproductive organs, which allows us to better distinguish between visitors and potential pollinators.

After camera removal, stigmas of the recorded flowers were collected and stored in ethyl alcohol. Germinated pollen tubes were stained and counted later in the lab using fluorescence microscopy following the methods described by Dafni *et al.* (2005) to see how changes in visitation frequency potentially caused by the experimental treatment affect the plant’s pollination success.

**Figure 2.**
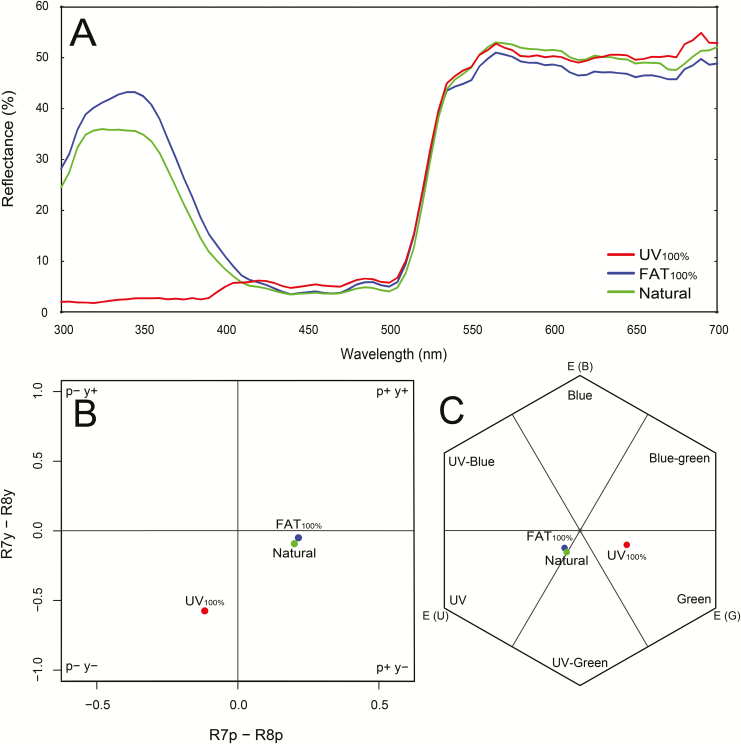
(A) reflectance of natural *H. camerooniana* flowers and those with experimental treatment and control (see Materials and Methods for more details). (B) Fly colour visual system displayed using the Troje model. (C) Bee colour visual system displayed using the colour hexagon model.

To check the floral UV pattern, UV photographs were taken using a Canon EOS 80D DSLR camera with a Helios 44-2 lens; UV conversion (i.e. replacing the internal hot mirror filter by a custom UV band pass filter) was done by LifePixel (Mukilteo, USA). During the picture taking, a 5-W UV flashlight was used for lighting (Fig. 1). To demonstrate the effect of experimental treatment, we measured reflectance of three flowers per treatment type (*FAT*_*100 %*_, *Natural control* and *UV*_*100 %*_) with 10 repeated measures per flower, using an Ocean Optics (Largo, USA) Jaz spectrometer. The graph depicts the mean of the repeated measures per treatment for the range of 300–700 nm (Fig. 2A). The bee and fly colour visual system was then mapped using the Troje model for flies (Troje 1993; Fig. 2B) and the colour hexagon for bees (Chittka 1992; Fig. 2C).

### Statistical analyses

To standardize the sampling effort (differences in flower longevity, as well as in the case of one or two flowers per experimental plant), all visits were transformed to visitation frequencies (no. of visits per hour and flower). This visitation frequency data (no. of visits per hour and flower) did not show normal distribution due to an overabundance of null values. In consequence, we used non-parametrical tests, being a permutational analogue of ANOVA and MANOVA in PRIMER 6 v. 6.1.13 and Permanova+ v. 1.0.3 (Anderson *et al.* 2008). *Post hoc* tests were used to compare the frequencies between the different treatments, with the recording day treated as random effect. Similarly, the effects of treatments on insect behaviour were tested by a permutational MANOVA. To check differences in amount of morphospecies and pollen tubes (i.e. the non-frequency data) we tried to implement generalized mixed-effect models, specifically Poisson, quasi-Poisson and zero-inflated distributions. However, due to a combination of the high overabundance of zero values and the negative values of the maximum likelihood estimations of the models, we were not able to apply these parametric methods and therefore, the non-parametrical tests (permutational analogue of ANOVA) were applied as well. The dependency of the number of pollen tubes on visitation frequency was tested by linear regression in STATISTICA (Statsoft, Inc 2011).

## Results

### Visitors of *H. camerooniana*

**Figure 3.**
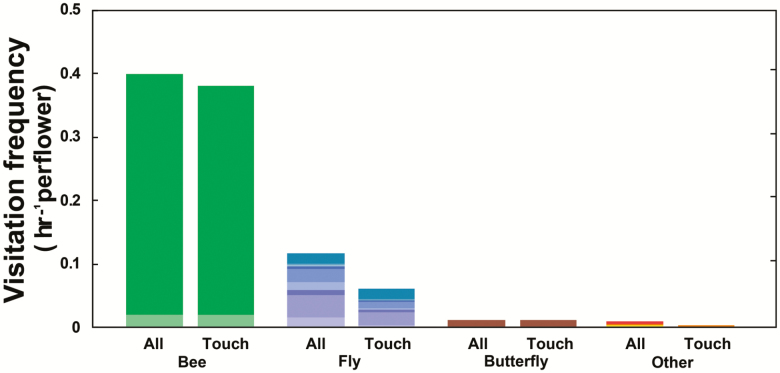
Frequency of all *H. camerooniana* flower visitors and visitors in contact with reproductive organs during daytime. Different colours indicate different morphospecies.

Considering all 50 observed plants, a total of 281 visitors were recorded. During daytime the flowers were mostly visited by bees (192 visits) and flies (59 visits), the only other considerable group of visitors were skipper butterflies (four visits). All other visitors (five visits) were evaluated as accidental and thus merged (Fig. 3). All bee visitors were composed of a single abundant morphospecies of solitary bee (187 visits) and the substantially rarer honeybee (*Apis mellifera*; five visits). The less abundant flies were considerably more taxonomically diverse, compared to bees, with nine recognized morphospecies. Bees visited the studied flowers mainly during morning hours, whereas fly visitation was distributed throughout the day **[see****Supporting Information—Figs S2 and S3****]**. Contrastingly, night visitors were rare (21 visits by 10 morphospecies) and consequently, with much lower visitation frequencies **[see****Supporting Information—Fig. S1****]**. Based on contacts with reproductive organs (Fig. 3; Table 1) bees may be considered as the main pollinator.

**Table 1.** Proportion of visits during which bees and flies touched reproductive organs of *H. camerooniana*. See Materials and Methods for the description of treatments.

	Bees		Flies	
Treatment	Stigma	Anthers	Stigma	Anthers
*UV*_*100 %*_	85.7	92.9	0.0	57.1
*F*_*100 %*_	76.7	95.3	15.4	23.1
*UV*_*50 %*_	59.4	90.6	28.6	57.1
*F*_*50 %*_	84.8	97.0	47.6	57.1
*Natural*	74.3	95.7	45.5	63.6

### Effects of UV pattern on visitors

**Figure 4.**
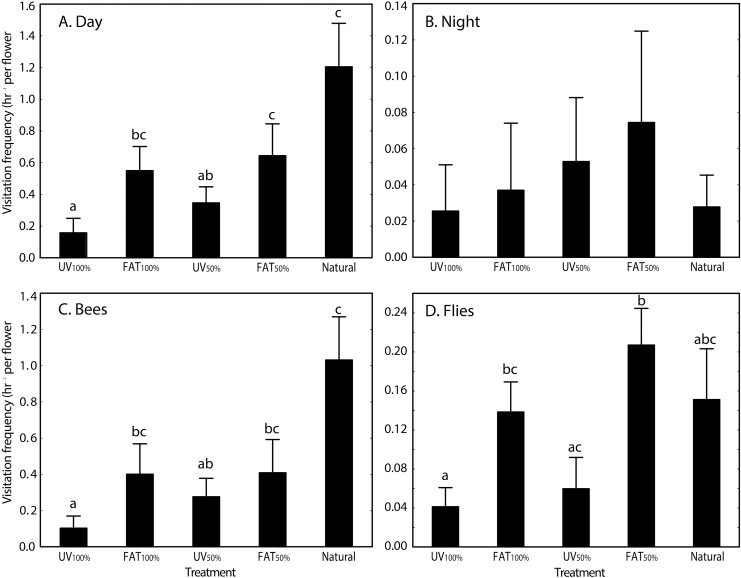
Effect of UV pattern manipulation on visitation frequencies of *H. camerooniana* for (A) day; (B) night; (C) bee; and (D) fly visitation during the day separately. Note: scaling of the *Y*-axis is not standardized due to the substantially lower number of visits between day and night, and between bees and flies. Means (bars) and SE (whiskers) are shown. The same letters above the columns indicate non-significant differences in the pairwise *post hoc* tests. See Materials and Methods for the description of treatments.

Individual treatments significantly affected visitation frequency during the day (*F*_ps_ = 6.71; df = 4; *P*_perm_ < 0.001). *UV*_*100 %*_ was significantly lower than all other treatments except *UV*_*50 %*_, which differed from *FAT*_*50 %*_ and *Natural control*. The highest visitation frequency was observed on untreated plants, but these did not significantly differ from the other two control treatments (*FAT*_*100 %*_, *FAT*_*50 %*_; Fig. 4). During the night, there was no significant treatment effect on frequency of flower visitors (*F*_ps_ = 0.36; df = 4; *P*_perm_ = 0.851). Visitation frequency was significantly affected by the treatment for both bees (*F*_ps_ = 6.13; df = 4; *P*_perm_ < 0.001) and flies (*F*_ps_ = 3.92; df = 4; *P*_perm_ = 0.009). In both visiting groups, *FAT*_*100 %*_ and *FAT*_*50 %*_ treatment has a significantly higher frequency than *UV*_*100 %*_, but *UV*_*50 %*_ was significantly lower than *FAT*_*50 %*_ for flies only (Fig. 4). The non-treated control (*Natural control*) significantly differed from UV-manipulated plants for bees only (Fig. 4). There was no significant effect of treatment on the number of morphospecies observed on the flowers (*F*_ps_ = 2.08; df = 4; *P*_perm_ = 0.103).

### Effects of treatments on visitor behaviour

**Figure 5.**
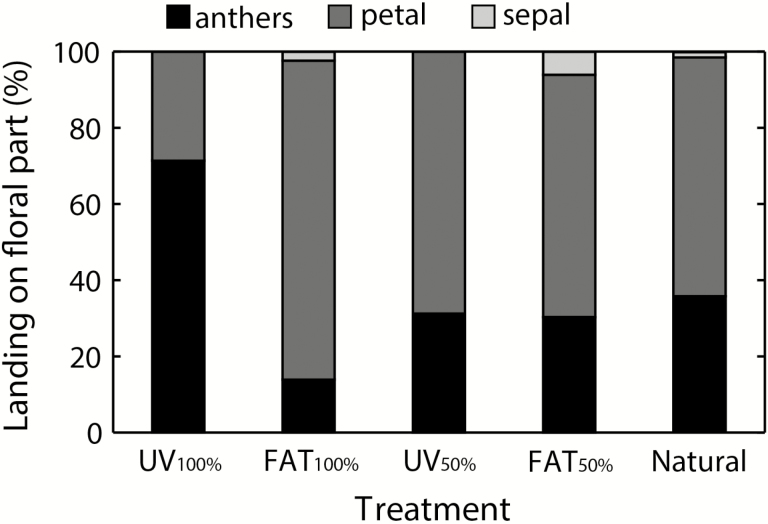
Effect of UV pattern manipulation on bee landing behaviour of *H. camerooniana* flowers. See Materials and Methods for the description of treatments.

We found a significant effect of treatment on bee landing behaviour (*F*_ps_ = 5.04; df = 4; *P*_perm_ = 0.004), but not on fly landing (*F*_ps_ = 1.08; df = 4; *P*_perm_ = 0.373). On *UV*_*100 %*_-treated flowers, bees landed mostly on anthers, whereas in other treatments bees usually landed on the petals before moving to anthers and stigma (Fig. 5). When collecting pollen, bees usually touched both anthers and stigmas, whereas flies had considerably fewer contacts with the reproductive organs during their visits (Table 1). There was no significant effect of treatment on bees (*F*_ps_ = 0.49; df = 4; *P*_perm_ = 0.770) and flies (*F*_ps_ = 0.44; df = 4; *P*_perm_ = 0.903) behaviour after landing. Bees spent 95 % of the flower visit duration by collecting pollen, while flies spent most time (68 %) crawling, sitting and flying between individual floral parts **[see****Supporting Information—Fig. S4****]**.

### Effect of treatment on the plant

**Figure 6.**
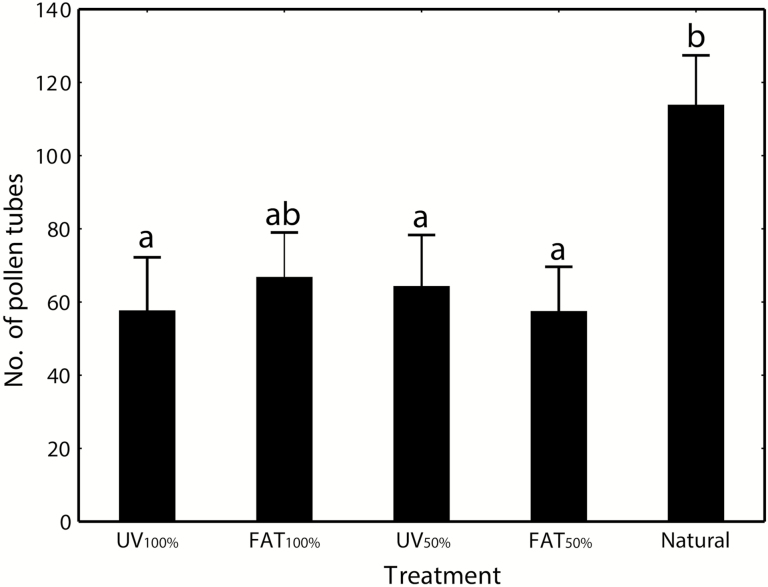
Effect of UV pattern manipulation on the number of germinated pollen tubes in stigmas of *H. camerooniana.* Means (bars) and SE (whiskers) are shown. The same letters above the columns indicate non-significant differences in the pairwise *post hoc* tests.

**Figure 7.**
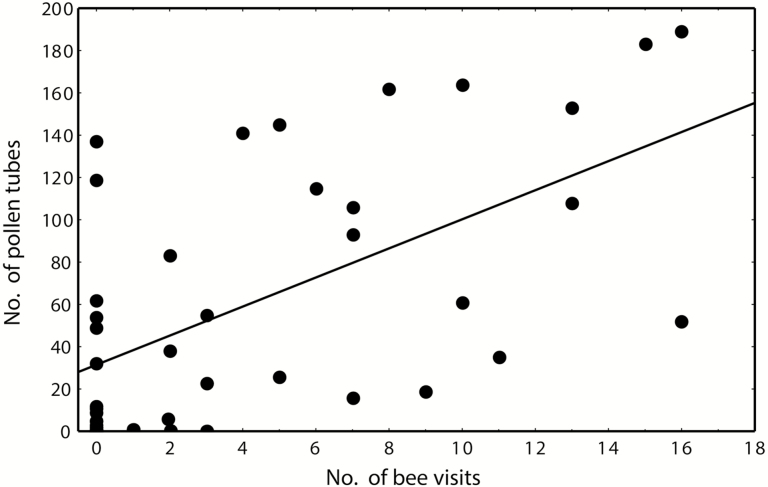
Effect of number of bee visits on number of germinated pollen tubes in stigmas of *H. camerooniana*.

The number of germinated pollen tubes significantly differed among treatments (*F*_ps_ = 3.66; df = 4; *P*_perm_ = 0.010), mainly due to a significantly higher number of pollen tubes germinated in non-manipulated flowers (Fig. 6). The pollen tube count increases with number of visits by bees (*r* = 0.57, *P* < 0.001; Fig. 7), but not of flies (*r* = −0.0073, *P* = 0.962).

## Discussion

Our study demonstrated that *H. camerooniana* is mainly pollinated by bees, confirming previous studies on pollination of *Hypoxis* plants (Singh 1999; Johnson and Andersson 2002), with the notable exception of *H. decumbens*, pollinated by flies in Venezuela (Raimúndez and Ramirez 1998). In addition to the previous studies of the genus based on visitation rates, we have confirmed that bees are the most efficient pollinators of *H. camerooniana* since: (i) bees were the most common visitors; (ii) in a large percentage of visits they are in contact with reproductive organs, which is expectable for these voracious pollen feeders; and (iii) their visits significantly increased numbers of germinated pollen tubes in stigmas. Contrary to Johnson and Andersson’s (2002) observations on *H. hemerocallidea* in South Africa, there were only few visits of honeybees compared to the abundant visits by a single morphospecies of small solitary bees. Moreover, honeybees seemed to be mostly searching for nectar, although we did not observe any nectar in flowers of *H. camerooniana*, consistent with other *Hypoxis* species (Johnson and Andersson 2002; Rudall 2002; Ren *et al.* 2019).

### UV and visitor frequency

The UV signal of flowers influenced the particular visitor frequency in different ways. Bees visited flowers more often when at least half of the petals reflected UV. However, although not significant, even the control flowers treated with non-absorbing cream differed in bee (but not fly) visitation frequencies from untreated flowers. The drop in visitation frequency between the treatments and their respective controls is consistent with the previous study of *H. hemerocallidea* (Johnson and Andersson 2002) in which fewer honeybees (*A. mellifera*) visited flowers with the floral UV reflectance obscured. While in alpine communities of New Zealand, Campbell *et al.* (2010) found experimentally manipulated flower colour, and not UV reflectance, to be more important for the visitation rates, other studies on UV pollinator visitation preferences showed similar results to ours (Peter and Johnson 2008; Rae and Vamosi 2013; Horth *et al.* 2014; Koski and Ashman 2014). Ultraviolet reflectance was revealed as highly important for bees pollinating *Eulophia zeyheriana* (Orchidaceae; Peter and Johnson 2008), whereas the general visitation rates of various visitors declined after manipulation of UV reflectance in two *Rudbeckia* species (Horth *et al.* 2014), *Mimulus guttatus* (Phrymaceae; Rae and Vamosi 2013), and *Argentina anserine* (Rosaceae; Koski and Ashman 2014). Therefore, UV reflectance plays an important role in pollinator attraction, but it can differ among flowering species, since other floral traits, such as scent, shape and colour, could be equally important.

Additionally, we have shown that having at least some UV reflectance is more important for selection of *H. camerooniana* flowers by bees than its UV pattern (*sensu*Koski and Ashman 2014), as flowers with fully covered petals by the UV-absorbing cream differed in bee visitation from those with all petals fully reflecting UV. Flies, however, although also showing a higher visitation frequency when the flower is fully or partially reflecting UV, did not significantly differentiate between *UV*_*100 %*_ and the *Natural control*. These results are discordant with the previous study of yellow UV-reflecting flowers of *A. anserine* (Koski and Ashman 2014) which demonstrated that the presence of UV pattern increased visitation by both bees and syrphid flies relative to both fully UV reflective or absorptive flowers. Nevertheless, bees were repeatedly described to prefer flowers with some colour pattern above the unicoloured ones (e.g. Waser and Price 1985; Papiorek *et al.* 2016).

It must be stipulated that UV reflectance is just one channel of communication of plants with insects (Chittka *et al.* 1994; Johnson and Andersson 2002). Additionally, other traits or factors need to be considered when looking at the pollination system. For example, the community context (Peter and Johnson 2008; Campbell *et al.* 2010), positioning of flowers and inflorescences (Lunau 1992b; Johnson *et al.* 2003a, b; Rae and Vamosi 2013; van der Kooi 2016), other optical principles of flower colouration (e.g. van der Kooi *et al.* 2014, 2016) and learning ability of visitors (Giurfa *et al.* 1995; Hammer 1997; Dyer *et al.* 2015). The learning ability of visitors is difficult to include into such field experimental studies. Supplementary controlled studies with naïve bees would be greatly beneficial to further disentangle the factors important in shaping the studied pollination system.

### UV and bee behaviour

The significant difference in the bee landing behaviour implies that the floral UV pattern can play an important role in orientation of bees on visited flowers. On the flowers completely covered with the UV-absorbing cream, bees mostly landed directly on the anthers and immediately started to collect pollen, whilst they landed mostly on petals of the flowers that at least partly reflected UV (i.e. all other treatments). This has proven that a disturbance of the UV pattern may change bees’ behaviour. Likewise, other colour patterns, such as floral guides or bullseye patterns, are considered to increase the plants’ reproductive success by helping pollinators to orientate to the flower centre (Waser and Price 1985; Dinkel and Lunau 2001; Leonard and Papaj 2011; Papiorek *et al.* 2016). However, bees actually make their first antennal contact preferably at the UV-absorbing floral area, irrespective of its spatial position within a flower (Papiorek *et al.* 2016). Therefore, one would expect bee visitors of *H. camerooniana* to prefer the centre of flowers with the UV-absorbing anthers, which is not the case. We thus hypothesize that in *H. camerooniana*, the UV-reflecting petals probably act as a landing platform, making flowers more visible for potential pollinators in its typical habitat of burnt montane grasslands, since the general UV reflection of similar grasslands vegetation is low (<5 %; Caldwell *et al.* 1983).

### Methodological biases of UV manipulation

When Johnson and Andersson (2002) used the genus *Hypoxis* for the experimental manipulation of floral UV reflectance to study the response of insect pollinators, they did not include the experimentally untreated plants (*Natural control*). They thus did not control for the effect of experimental manipulation on natural insect behaviour. In our experiment, which based the methodology largely on the referred study, we demonstrated that such experimental setting is useful to investigate the effect of floral UV signalling on visitors. But at the same time, we discovered that experimental controls (i.e. flowers covered by the non-UV-absorbing cream compound; *FAT*_*100 %*_, *FAT*_*50 %*_) can differ from the untreated natural flowers. The experimental controls showed lower (but not statistically significant) visitation rates than the natural control for bees. Furthermore, the numbers of germinated pollen tubes on stigmas of natural control flowers of *H. camerooniana* were significantly higher compared to all treated flowers, apart from the control with fully covered non-UV-absorbing cream. These lower visitation rates and lower number of germinated pollen tubes could be explained by several factors, e.g. less evaporation of scents or changes in the glossiness of the flower. It also proved that we did not cause pollination during handling of experimental flowers.

Additionally, we showed that this effect can be visitor-specific. Flies, generally a more olfactory-oriented group than bees (Roy and Raguso 1997), were not affected by the experimental controls at all. For this group, the UV manipulation treatments (*UV*_*100 %*_, *UV*_*50 %*_) did not significantly differ from the *Natural control*. Although, they were visited by significantly less flies than the controls (*FAT*_*100 %*_, *FAT*_*50 %*_). We thus speculate that the effect of UV manipulation is at least partly compensated by the potential attraction of Muscidae and Sarcophagidae flies (which created most of the diversity of recorded fly visitors) to the duck preen gland fat. Bees, as generally more visually oriented insects, expressed higher, but still non-significant, differences among natural and experimental controls. Furthermore, the thickness of the cream layer or amount of cream in the treatments has not been considered in our study but might play a role in the attraction of flies.

In summary, although the duck preen gland fat is a commonly used vector for the UV-manipulating agents (e.g. Johnson and Andersson 2002; Peter and Johnson 2008; Welsford and Johnson 2012; Rae and Vamosi 2013), it affects natural insect behaviour. Consequently, we strongly encourage ‘calibration’ of results by controlling for the chemical vector’s (duck preen gland fat in our case) effects in similar experimental studies.

## Conclusion

The primary pollinators of *H. camerooniana* in the Afromontane grasslands of Mount Cameroon were bees. When UV reflectance was completely removed visitation rates of bees decreased, whereas the decrease of frequency on half-treated flowers was not significant (although it decreased as well when considering all daytime visitors). The complete UV reflectance removal changed the landing behaviour of bees as well, confirming that altering the natural UV patterns affects both visitation rates and behaviour.

Furthermore, based on our results we also encourage the inclusion of a natural control in the experimental designs of similar manipulative studies to control for the substances used in floral manipulation.

## Supporting Information

The following additional information is available in the online version of this article—

**Figure S1.** Frequency of nocturnal visits of *Hypoxis camerooniana* flowers.

**Figure S2.** Diurnal changes in bee visitation frequencies on flowers of *Hypoxis camerooniana* after manipulation of their ultraviolet (UV) reflectance. Means (bars) and SE (whiskers) are shown.

**Figure S3.** Diurnal changes in fly visitation frequencies on flowers of *Hypoxis camerooniana* after manipulation of their ultraviolet (UV) reflectance. Means (bars) and SE (whiskers) are shown.

**Figure S4.** Bee (A) and fly (B) behaviour on flowers of *Hypoxis camerooniana* after manipulation of their ultraviolet (UV) reflectance. There was no significant effect of treatment on both bee (*F*_ps_ = 0.49; df = 4; *P*_perm_ = 0.770) and fly (*F*_ps_ = 0.44; df = 4; *P*_perm_ = 0.903) behaviour after landing.

plz057_suppl_Supplementary_Data

plz057_suppl_Supplementary_Material

## Data

An excel file with the data used for analyses and graphs is available in the online version of this article.

## Sources of Funding

This project was funded by the Czech Science Foundation (16‐11164Y), the Grant Agency of the Charles University (GAUK No. 356217) and the Charles University (PRIMUS/17/SCI/8 and UNCE204069).

## Contributions by the authors

All authors helped design the experiment, Y.K., R.D.K., M.B., J.E.J.M. and S.J. were involved in data collection; R.D.K., S.J., R.T. and Y.K. were in charge of data analyses and writing first drafts of the manuscripts. All authors contributed to writing and editing of the manuscript.

## Conflict of Interest

None declared.
