# Supplementary material for: The role of ultraviolet reflectance and pattern in the pollination system of Hypoxis camerooniana (Hypoxidaceae)
Source: AoB Plants. 2019 Sep 23;11(5):plz057. doi: 10.1093/aobpla/plz057 (PMC6803167; doi:10.1093/aobpla/plz057)
Supplement: plz057_suppl_Supplementary_Material [file plz057_suppl_supplementary_material.pdf]

Supplementary material

Article title: The role of ultraviolet reflectance and pattern in the pollination system of *Hypoxis camerooniana (Hypoxidaceae).*

The following supplementary material is available for this article:

Fig. S1: Frequency of nocturnal visits of *Hypoxis camerooniana* flowers.

Fig. S2: Diurnal changes in bee visitation frequencies on flowers of *Hypoxis camerooniana* after manipulation of their UV reflectance. Means (bars) and SE (whiskers) are shown.

Fig. S3: Diurnal changes in fly visitation frequencies on flowers of *Hypoxis camerooniana* after manipulation of their UV reflectance. Means (bars) and SE (whiskers) are shown.

Fig. S4: Bee (A) and fly (B) behaviour on flowers of *Hypoxis camerooniana* after manipulation of their UV reflectance. There was no significant effect of treatment on both bee (F_{ps}=0.49; df=4; P_{perm}=0.770) and fly (F_{ps}=0.44; df=4; P_{perm}=0.903) behaviour after landing.

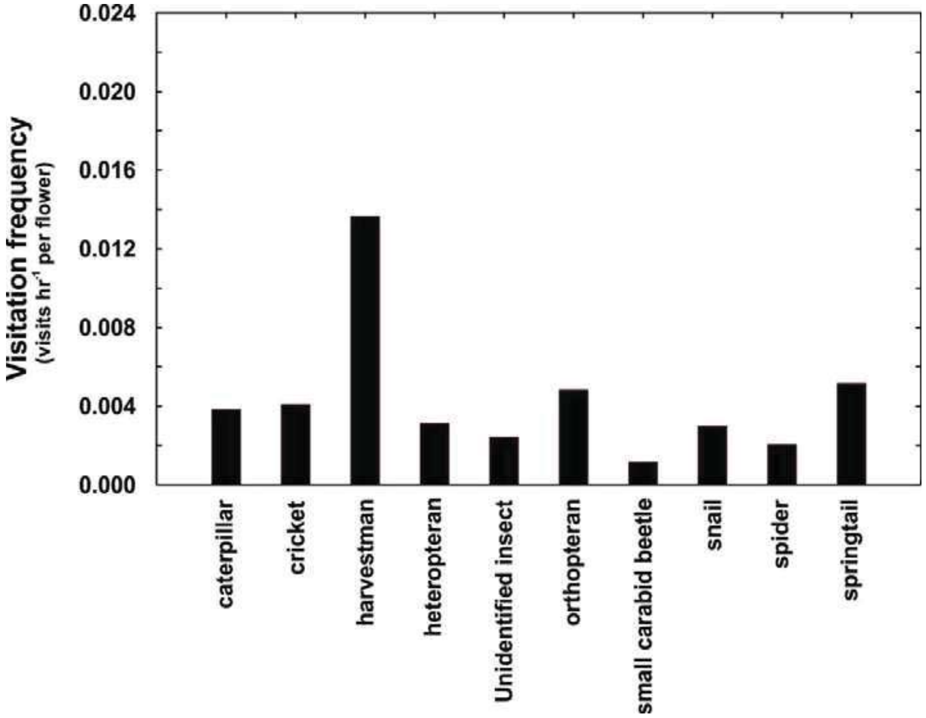

Fig. S1: Frequency of nocturnal visits of *Hypoxis camerooniana* flowers.

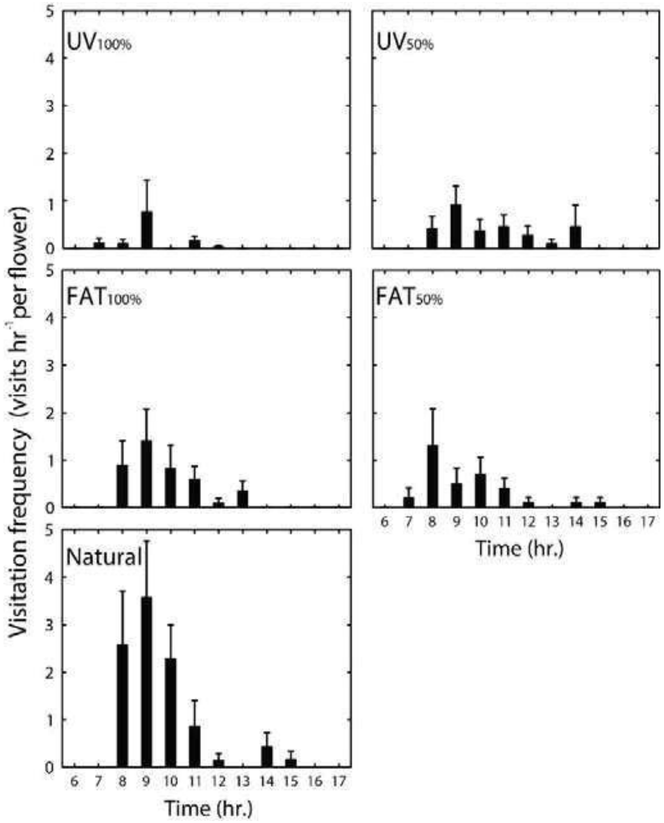

Fig. S2: Diurnal changes in bee visitation frequencies on flowers of *Hypoxis camerooniana* after manipulation of their UV reflectance. Means (bars) and SE (whiskers) are shown.

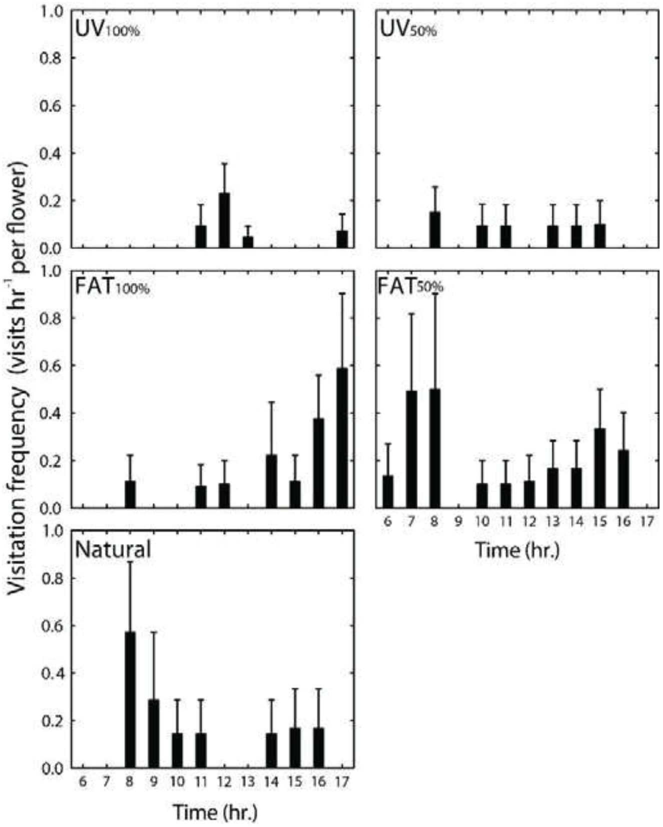

Fig. S3: Diurnal changes in fly visitation frequencies on flowers of *Hypoxis camerooniana* after manipulation of their UV reflectance. Means (bars) and SE (whiskers) are shown.

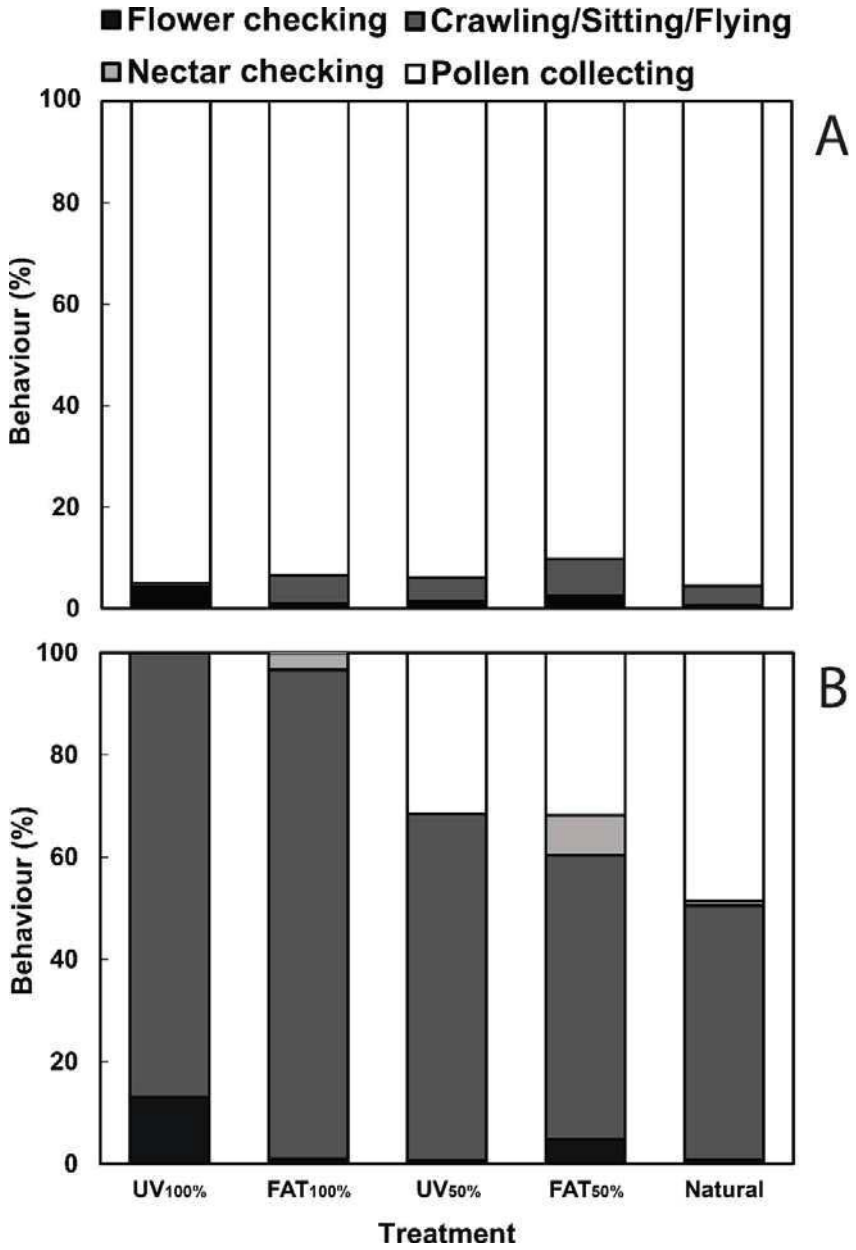

Fig. S4: Bee (A) and fly (B) behaviour on flowers of *Hypoxis camerooniana* after manipulation of their UV reflectance. There was no significant effect of treatment on both bee (F_{ps}=0.49; df=4; P_{perm}=0.770) and fly (F_{ps}=0.44; df=4; P_{perm}=0.903) behaviour after landing.
